# Incidence and treatment approach of intraocular pressure elevation after various types of local steroids for retinal diseases

**DOI:** 10.1007/s00417-023-06163-5

**Published:** 2023-07-11

**Authors:** Agata Anna Wykrota, Alaa Din Abdin, Cristian Munteanu, Ursula Löw, Berthold Seitz

**Affiliations:** Department of Ophthalmology, Saarland University Medical Center (UKS), Homburg/Saar, Germany

**Keywords:** Macular edema, Steroidal agents, Ocular hypertension, Secondary ocular hypertension

## Abstract

**Purpose:**

For the treatment of macular edema, in addition to the use of antivascular endothelial growth factors, steroids are also used intravitreally and sub-Tenon. Side effects include among others cataract formation and elevation of intraocular pressure (IOP). The aim of this retrospective study was to elicit the IOP elevation after administration of various steroidal medication, the time of onset, and the efficacy of the administered IOP-lowering therapies.

**Methods:**

We included 428 eyes with a postoperative (*n* = 136), diabetic (*n* = 148), uveitic macular edema (*n* = 61), and macular edema after retinal vein occlusion (*n* = 83). These patients were treated with one or more diverse steroidal agents once or multiple times. These drugs included: triamcinolone acetonide (TMC) as intravitreal injection (TMC IVI) or sub-Tenon (TMC ST), as well as dexamethasone (DXM) and fluocinolone acetonide (FA) intravitreally. An increase of IOP of ≥ 25 mmHg was designated as pathological. A steroid response in anamnesis, the time of onset of IOP rise from the first administration, and the therapy administered were documented.

**Results:**

Of 428 eyes, 168 eyes (39.3%) had IOP elevation up to a mean of 29.7 (SD ± 5.6) mmHg, which occurred at a median of 5.5 months. Steroids most frequently leading to rise of IOP included DXM (39.1% of all eyes receiving that drug), TMC IVI (47.6%), TMC ST combined with DXM (51.5%), DXM with FA (56.8%), and TMC IVI with DXM (57.4%). A Kaplan–Meier analysis and the Log Rank test showed a significant difference (*p* < 0.001). IOP rise was treated as follows: 119 conservatively (70.8%), and 21 surgically (12.5%, cyclophotocoagulation 8.3%, filtering surgery 1.8%, in 4 the steroidal drug implant was removed 2.4%), and 28 eyes received no therapy (16.7%). Sufficient IOP regulation was achieved in 82 eyes (68.9%) with topical therapy. In 37 eyes (31.1%) with persistently elevated intraocular pressure, topical therapy had to be continued over the follow-up of 20 ± 7 months.

**Conclusions:**

IOP increases after any type of steroid application are not rare. Results of our study let us suspect that especially therapy with intravitreal dexamethasone, either as a monotherapy or in combination with another steroid, tends to increase IOP more than other steroids. Regular IOP checks are necessary after each steroid administration, with possible initiation of long-term conservative and/or surgical therapy if necessary.


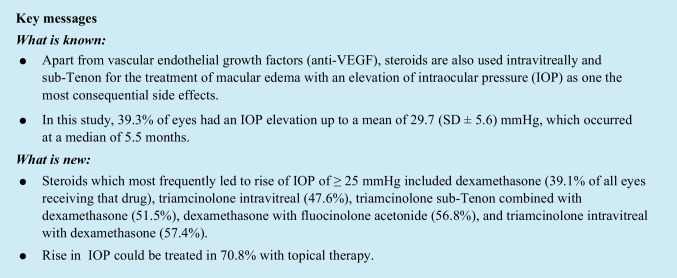


## Introduction

Glaucoma is considered to be the second leading cause of visual impairment and blindness worldwide, affecting about 70 million people [1]. The most common form of this disease, concerning 70% of cases, is primary open-angle glaucoma [2]. A major risk factor for the disease is an elevated intraocular pressure (IOP), although some cases present with normal IOP [3]. In any case, eyes with both high tension and normal tension glaucoma are characterized by a progressive loss of retinal nerve fiber cells with subsequent reduction of the visual field [2] and reducing IOP is considered an effective treatment [4]. Therapeutic use of glucocorticoids can cause an elevation of IOP called steroid-induced ocular hypertension (SIOH) and thereby steroid-induced – secondary – glaucoma (SIG) by initialing signaling cascades affecting expression of genes, which causes a highly personalized pharmacological response [5]. It was first in 1950 when an elevation of IOP was documented after systemic administration of adrenocorticotrophic hormone [6]. An increase of IOP after the topical administration of cortisone was noticed first in 1954 [7]. Since then, the phenomenon of SIOH and SIG has been studied intensively and risk factors, the pathophysiology, as well as treatment were investigated. Nowadays, steroidal drug implants are a known treatment option for retinal diseases, such as macular edema [8]. An increase in IOP under steroid therapy, resulting in a secondary glaucoma, can be observed in about 30% of the population. This phenomenon is called "steroid response". In about 5%, a "high-response" is present, with a pressure increase of more than 15 mmHg above the baseline pressure [9]. If the steroid-induced ocular hypertension resulting from the steroid therapy is of a significant magnitude, and left uncontrolled and untreated, it can lead to steroid-induced glaucoma with glaucomatous optic neuropathy [10].

There are many conditions leading to macular edema, four of which are mentioned in this paper: diabetes, retinal vein occlusion, inflammatory eye diseases (uveitis) and status post eye surgery. Although having various pathophysiology, they can be all treated with steroidal drugs.

The aim of this retrospective study was to elicit the intraocular pressure increases after steroid administration for retinal disease, the time of onset, as well as the efficacy of the administered eye pressure-lowering therapeutic approaches.

## Methods

### Study design and database

The data collection was performed retrospectively on 428 eyes of 349 patients in the period from 01.01.2016 to 31.08.2021. The study was performed in a single center, the Department of Ophthalmology at the Saarland University Medical Center (UKS) in Homburg/Saar. Patients were identified using the FIDUS (our electronic patient records) [11] and SAP (Systems, Applications and Products in data processing – our internal hospital information systems). Inclusion criterium for this study was a diagnosis of macular edema as a result of status post eye surgery, diabetic macular edema, retinal vein occlusion, and uveitis. Patients with confirmed primary open-angle glaucoma were included. Minors (patients < 18 years old), however, were excluded from the study. Performed surgeries leading to secondary macular edema included cataract surgery, pars plana vitrectomy (PPV), these two procedures combined, Descemet membrane endothelial keratoplasty (DMEK), and penetrating keratoplasty (PK). Retinal vein occlusion included both central (CRVO) and branch retinal vein occlusion (BRVO). Macular edema was diagnosed based on fundoscopy, optical coherence tomography and fluorescein angiography, which were performed before the first steroidal drug application. Macular edema was treated with steroidal agents once or multiple times, and with one or more steroids. If a subject presented with a steroid response, i.e. IOP rise, we documented the time after first steroid administration and the therapy administered. An IOP rise was described as ≥ 25 mmHg [12]. The therapies administered in our study were: (1) conservative with eye drops and (2) surgical, including cyclophotocoagulation, filtering surgery and removal of steroidal drug implant. Afterwards, we documented whether the chosen treatment was beneficial for the eye pressure or not. IOP of ≤ 20 mmHg was considered as well-regulated and the time after the introduction of therapy was documented. In case of conservative eye drop therapy, we also documented whether a patient was able to stop taking the medication.

This study and all investigational protocols were approved by the Saarland University Medical Center as well as the Ethics Committee of the Medical Association of Saarland, Germany (Nr. 123/20, date: 16.06.2020).

### Target figures

All patients had follow-ups for 24 months with visits at day 1 (first steroid administration), 6–8 weeks, 6, 12, 18 and 24 months.

### Ophthalmological examinations

A complete ophthalmological clinical evaluation performed for all patients included the best corrected visual acuity (BCVA), intraocular pressure (IOP) measurement using Goldmann applanation tonometry, and anterior and posterior segment examination with the slit lamp (Slit Lamp BX 900®, Haag-Streit, Köniz, Switzerland). Macular edema with its central macular thickness (CMT) was measured with macular optical coherence tomography (M-OCT) (Spectralis OCT, Heidelberg Engineering, Heidelberg, Germany). Fluorescein angiography (FA, Heidelberg Engineering, Heidelberg, Germany) was usually performed once at the initial presentation to confirm the diagnosis. If necessary – it was repeated.

### Application process

In this study, macular edema was treated with triamcinolone acetonide sub-Tenon (TMC ST) or intravitreally (TMC IVI), dexamethasone intravitreally (DXM) and fluocinolone acetonide intravitreally (FA) used in monotherapy or different combinations in our Macula Outpatient Department (IVI center [13]). Physicochemical characteristics of each drug are summarized in Table 1. Patients were examined before each steroidal drug application to check for possible contraindications to the injection, such as any kind of eye inflammation or other pathologies. We examined the BCVA, IOP, anterior and posterior ocular segment and educated the patients about the injection, possible risks and complications as well as had them sign the consent form. Afterwards, the eye to be injected was marked and dropped with tropicamide, phenylephrine, to dilate the pupil, and polyhexanide 0.02% eye drops, as an antiseptic. Altogether, the eye was dropped with polyhexanide twice before and once after an injection. The anamnesis and current therapy were also ascertained, to find out about possible changes in medication applied from an ophthalmologist or, for example, an elevated IOP that had to be treated. We performed an OCT to check the macular edema before the first injection and then according to the treatment plan every one, three or four appointments.

**Table 1:** Comparison of each steroid drug with its active substance, application form, time of action, maximal concentration (if available), concentration at the end of action time (if available), its approved and off label use [12, 15–24]

Trade name	Active substance	Applica-tion	Time of action	Maximal concentration (day/value)	Concentration at the end of action time (day/value)	Approved use
VOLON A	Triamcinolone acetonide	Intravitreal injection	12 weeks to couple of months	2.15 to 7.20 μg/ml		-
VOLON A	Triamcinolone acetonide	Sub-Tenon injection	12 weeks	NI		-
OZURDEX	Dexamethasone	Intravitreal implant	6 months	0.094 ng/ml	0.05 ng/ml	DME RVO Uveitis
ILUVIEN	Fluocinolone acetonide	Intravitreal implant	36 months	7^th^ day	100 pg/mL	DME Uveitis

*DME* = diabetic macular edema, *RVO* = retinal vein occlusion, *PME* = postoperative macular edema, *NI* = no information

We applied triamcinolone acetonide, dexamethasone and fluocinolone acetonide intravitreally and triamcinolone acetonide sub-Tenon under a drop/gel anesthesia with proparacaine eye drops and xylocaine gel for at least 10 min before the injection. 0.05 ml of TMC, 700 μg of DXM or 190 μg of FA was entered tempo-inferiorly with displacement of the conjunctiva at 4 mm from the limbus. Afterwards, the light perception was checked and when positive – the patient was released from our outpatient department. After the steroid application intravitreally, we always recommended a check-up appointment in the next two or three days at our department or at external doctors´ offices. A dose of 0.15 ml of TMC was administered sub-Tenon under the tempo-inferior conjunctiva in drop/gel anesthesia with proparacaine eye drops and xylocaine gel for at least 10 min before the application.

For DME and macular edema after vein occlusion, we administered one TMC IVI as standard, and if no complications occurred, we performed intravitreal injection of DXM after at least 8 weeks. DXM was usually applied every 4–6 months, while FA every 2–3 years. In the absence of improvement after topical and systemic therapy for UME (uveitic macular edema), TMC sub-Tenon followed by intravitreal injection of DXM was administered. Postoperative macular edema was principally treated with TMC sub-Tenon [14]. No standard postoperative therapy was administered, especially no antibiotics or steroids.

### Therapy of steroid-induced ocular hypertension applied in this study

The first-choice intraocular pressure-lowering therapy applied in our department were topically administered drugs. The recommended treatment mostly included alpha-2 (α_2_) adrenergic receptor agonists 2 or 3 times per day, carbonic anhydrase inhibitors 2 times per day, beta (β-) blockers 2 times per day as single medication, or as combination medication. If the IOP was not low enough, we prescribed acetazolamide 250 mg in doses of 0.5, 1, 2 or 3 per day, according to needs of the patient, administered orally. Prostaglandin analogues were not recommended as they might trigger an anterior chamber inflammation and macular edema, as well as its progression through arachidonic acid and pro-inflammatory process activation [25]. At first, we prescribed a therapy with one medication (= monotherapy). If the drug was not tolerated it was switched. In contrast, if the target IOP could not be reached – another drug was added. If a patient still presented with increased IOP, we decided to run a surgical, IOP lowering intervention.

Procedures carried out in our study included a cyclophotocoagulation, trabeculectomy and vitrectomy to remove a steroidal drug implant and ensure a normalization of an elevated IOP. Anesthesia applied to patients in our study included a topical, retrobulbar, sub-Tenon and general anesthesia.

### Statistics

Data was collected in a Microsoft Excel 365 spreadsheet. Descriptive analysis of the patient collective was already possible using Excel. Absolute (number of cases) and relative frequencies (percentage) were calculated. In addition, maximum, minimum, mean, and standard deviation could be calculated for some parameters to better describe the patient groups. The arithmetic mean was always calculated and documented as the mean value.

For the statistical analysis we used IBM SPSS Statistics v27. The Pearson Chi-Squared test was used to compare categorical and ordinal variables as well as to show a significant association between groups and categories. For the comparison of the continuous variable over time between numbers of categories the General Linear Model was used. Also, the pairwise comparison between time points was adjusted with Bonferroni correction. A p-value of < 0.05 was considered to show a significant result.

Both eyes were included in 79 patients of 349 (22.6%), therefore the interclass correlation coefficient (ICC) [26] between the left and right eye of the same patient was calculated for all parameters to exclude a significant effect due to correlated data.

## Results

### Study collective

#### Demographic data, general and eye anamnesis (Table 2)

**Table 2:** Demographic data, general and eye anamnesis of 349 patients

Demographic data and eye anamnesis	
Number of patients	Total 349 Male 188 (53.9%) Female 161 (46.1%)
Average age	68.4 ± 11.9 years
Hypertension	251 (71.9%)
Diabetes mellitus	146 (41.8%)
Anticoagulation	159 (45.6%)
Number of eyes	428
Right eyes	236 (55.1%)
Left eyes	192 (44.9%)
Eyes with primary open-angle glaucoma	65 (15.2%)
Eye pressure lowering eye drops	58 (13.6%)
Positive family history of glaucoma	7 (2%)
Amblyopic eyes	14 (3.3%)
History of trauma	8 (1.9%)
Status post cataract surgery	328 (76.6%)
Status post pars plana vitrectomy	236 (31.5%)

The study included 428 eyes of 349 patients, of which 188 (53.9%) were male and 161 (46.1%) were female with an average age of 68.4 (± 11.9). The general anamnesis showed that 251 (71.9%) subjects presented with hypertension, 146 (41.8%) with diabetes mellitus with an average HbA1c of 7.5% and 159 (45.6%) took an anticoagulant at that time.

Out of 428 eyes, we counted 236 (55.1%) right eyes and 192 (44.9%) left ones. The p-value of ICC for all main outcome measures between left and right eyes was > 0.35, which indicated a non-significant correlation between the two eyes in examined patients. According to the eye anamnesis, 65 (15.2%) eyes presented with primary open-angle glaucoma using only one ocular pressure lowering eye drop, out of which 7 (2%) had a positive family history of glaucoma. 14 eyes were amblyopic (3.3%), 8 eyes had a history of trauma (1.9%), 328 eyes (76.6%) had undergone cataract surgery previously and 135 eyes (31.5%) pars plana vitrectomy.

All eyes that experienced trauma (*n* = 8, 1.9%) suffered from a postoperative macular edema later on.

#### Classification according to diagnosis

We included 136 eyes (31.8%) with a postoperative macular edema (PME), 148 (34.6%) with diabetic macular edema (DME), 83 (19.4%) with macular edema after a retinal vein occlusion (RVO, both central retinal vein occlusion – CRVO, and branch retinal vein occlusion – BRVO) and 61 (14.3%) with uveitic macular edema (UME).

#### Classification according to steroids administered

As far as administration of single drug and drug combinations was concerned, the exact data is presented in Table 3.

**Table 3:** Elevation of intraocular pressure in eyes treated with monotherapy or combination of steroidal agents (TMC ST = triamcinolone sub-Tenon; FA = fluocinolone acetonide; DXM = dexamethasone; TMC IVI = triamcinolone intravitreal)

Steroidal drug as monotherapy and drug combination	Frequency (n, % of all drug combinations)	IOP elevation (n, % of each drug combination)	Time until IOP rise in median (months)
TMC ST	130 (30.4%)	30 (23.1%)	5
FA	16 (3.7%)	5 (31.3%)	11
DXM	133 (31.1%)	52 (39.1%)	5
TMC IVI	21 (4.9%)	10 (47.6%)	1
TMC IVI + DXM	47 (11%)	27 (57.4%)	7
DXM + FA	37 (8.7%)	21 (56.8%)	6
TMC ST + DXM	33 (7.7%)	17 (51.5%)	4
TMC IVI + DXM + FA	6 (1.4%)	4 (66.7%)	8
TMC IVI + TMC ST + DXM	3 (0.7%)	1 (33.3%)	8
TMC ST + DXM + FA	1 (0.2%)	1 (100%)	8
TMC IVI + TMC ST	1 (0.2%)	0 (0.0%)	8

### Period of follow-up

A follow-up at 6–8 weeks was done for all the patients, 386 (90.2%) patients were controlled after 6 months, 323 (75.5%) of them had a follow-up at 1 year, 256 (59.8%) of them came in for a control visit after 18 months, while a full follow-up of 24 months was reached by 220 patients (51.4%). We considered the unscheduled visits as well, as the patients were often referred from a doctor because of problems after steroid administration, mostly because of IOP elevation.

### Visual acuity (VA)

The BCVA improved in patients with postoperative macular edema with an average of 0.26 (± 0.2) decimal before the first steroid application, 0.4 (± 0.26) after 12 months and 0.4 (± 0.26) after 24 months of follow-up, as well as in patients with uveitic macular edema with 0.25 (± 0.13) preoperatively, 0.37 (± 0.2) after 12 months and 0.42 (± 0.28) after 24 months. Subjects suffering from diabetic macular edema had an average BCVA of 0.43 (± 0.22) before the steroid application, 0.46 (± 0.25) after 12 months and 0.38 (± 0.25) after 24 months. Patients after a retinal vein occlusion presented with an average BCVA of 0.34 (± 0.2) preoperatively, 0.37 (± 0.24) after 12 months and 0.31 (± 0.26) after 24 months.

Statistical analysis showed no significant difference between BCVA in eyes with an elevated IOP as opposed to eyes without an IOP elevation. VA in 168 eyes with an elevated IOP was as follows: 0.34 (± 0.21) decimal before the first steroid application, 0.4 (± 0.25) after 12 months and 0.35 (± 0.24) after 24 months of follow-up.

### Central macular thickness (CMT)

As far as the CMT is concerned, it was reduced in all groups under the steroid therapy. It amounted on average 465 μm before the first steroid administration, 380 μm after 6–8 weeks, 384 μm after 6 months, 382 μm after 12 months, 370 μm after 18 months, and 369 μm after 24 months. Regarding CMT in different groups before the steroid application and at last control of 24 months, we measured 504 μm (± 109) and 384 μm (± 105) in the postoperative group, 420 μm (± 119) and 362 μm (± 97) in diabetic patients, 429 μm (± 129) and 386 μm (± 144) in subjects after retinal vein occlusion and 497 μm (± 108) and 356 μm (± 125) at uveitic patients.

### Intraocular pressure (IOP)

An IOP was measured at every visit, with an average of 14.3 (SD ± 3.3) mmHg before the first steroid administration, 15.7 (SD ± 4.6) mmHg after 6–8 weeks, 15.8 (SD ± 4.6) mmHg after 6 months, 15.3 (SD ± 4.0) mmHg after 12 months, 15.2 (SD ± 4.6) mmHg after 18 months, and 15.5 (SD ± 5.4) mmHg at 24 months.

### Steroid response

If a subject presented with an IOP rise of ≥ 25 mmHg, we documented the time after first steroid administration and the therapy administered. Out of a total of 428 eyes, 168 presented with an IOP elevation of ≥ 25 mmHg, with a mean value of 29.7 (SD ± 5.6) mmHg. The rise of ocular pressure occurred on average after 7.5 months (SD ± 6.2) from the first steroid administration, with a median of 5.5 months. Subjects presented with an elevated ocular pressure of ≥ 25 mmHg after a minimum of 2 weeks and a maximum of 24 months.

**Fig. 1:**
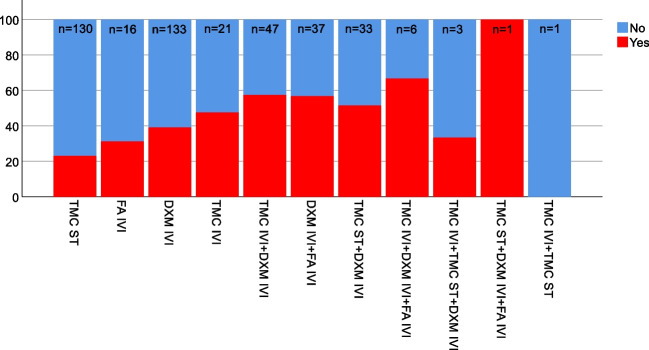
Elevation of intraocular pressure in eyes treated with monotherapy or combinations of steroidal medication (TMC ST = triamcinolone sub-Tenon; FA = fluocinolone acetonide; DXM = dexamethasone; TMC IVI = triamcinolone intravitreal)

**Fig. 2:**
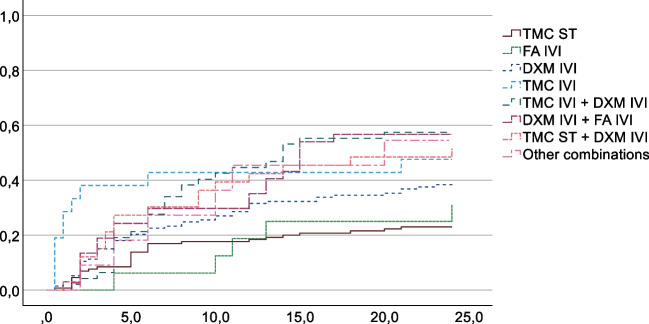
Median time of an IOP elevation (in months) after the first steroidal agent application in monotherapy, most applied steroid combinations, and the rest of steroid combinations (TMC ST = triamcinolone sub-Tenon; FA = fluocinolone acetonide; DXM = dexamethasone; TMC IVI = triamcinolone intravitreal)

When it came to the rise of IOP after administration of steroids in a monotherapy or as a drug combination, steroids mainly leading to a rise in IOP included DXM (39.1% of all eyes receiving that drug), TMC ST (23.1%), TMC IVI combined with DXM (57.4%), DXM with FA (56.8%), and TMC ST with DXM (51.5%) (Table 3 and Fig. 1). Kaplan–Meier analysis and the Log Rank test showed a significant difference (*p* < 0.001) in between types of steroidal medication used and the moment the patient had an IOP elevation. IOP rise was documented after a median of 1 month in case of TMC IVI and up to 11 months after application of FA (Fig. 2). Additionally, 57.7% out of all eyes with an IOP elevation were treated with a steroidal drug monotherapy. Furthermore, the steroid combinations similarly led to the IOP increase. Seventy-one eyes (42.3%) were treated with steroid combinations that resulted in IOP elevation in 51 to 57% of eyes. The most used combinations included TMC ST with DXM, DXM with FA, and TMC IVI with DXM.

#### Glaucoma patients before steroid application

As far as eyes with primary open-angle glaucoma are concerned, we noted 65 of them. 30 (46.2%) had no IOP elevation after application of a steroid agent and 35 (53.8%) presented an increased IOP, which makes 20.8% of all 168 eyes with an elevated IOP in this study. Thus, eyes with previous glaucoma had a significantly (*p* < 0.009) increased risk of developing an IOP elevation after steroid application.

#### Rubeosis iridis

Rubeosis iridis, as an additional parameter that may cause a rise in ocular pressure, was documented in one patient before the steroid therapy and additionally in two patients after 6 months. The first patient presented with a diabetic macular edema and proliferative diabetic retinopathy; she also had an IOP elevation of 40 mmHg maximum. The second subject with the same diagnosis additionally had an ischemic maculopathy, and IOP rose to more than 25 mmHg. The third patient, who developed rubeosis iridis after 6 months, had uveitis intermedia with vasculitis, with IOP elevations of up to 50 mmHg.

#### Lens status – IOP elevation in phakic versus pseudophakic eyes

**Fig. 3:**
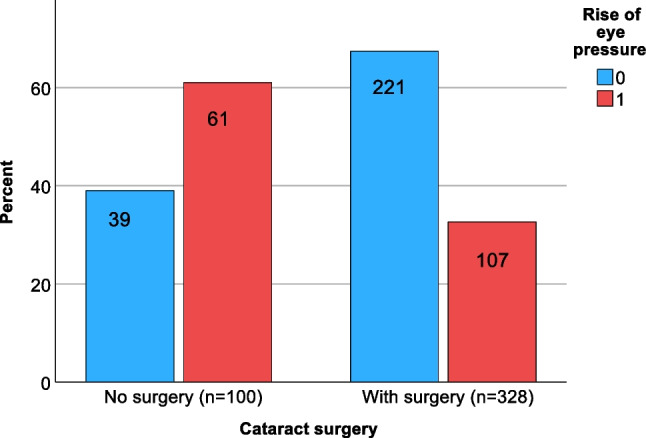
Percentage of eyes with and without IOP elevation in phakic and pseudophakic eyes (0 = no rise of IOP; 1 = rise of IOP)

Lens status showed to be statistically significant (p < 0.01) as far as rise of IOP is concerned. Out of 100 phakic eyes in general population, 61% (*n* = 61) showed an elevated IOP after steroid application. In comparison, IOP elevation was documented only in 32.6% (*n* = 107) of pseudophakic eyes (*n* = 328) (Fig. 3). Thus, lens status could be a predictive factor for IOP elevation after steroid administration.

**Fig. 4:**
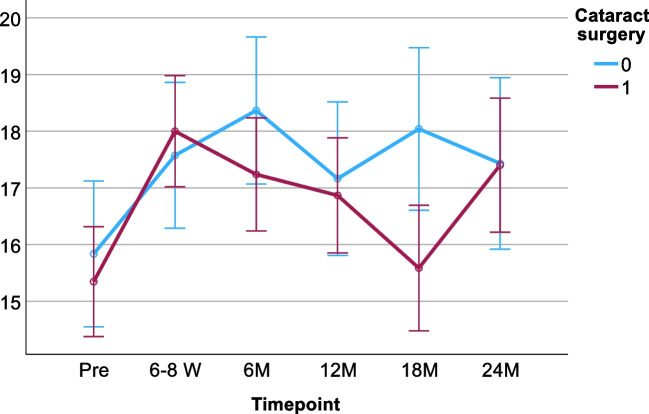
Average IOP in phakic and pseudophakic eyes (0 = no cataract surgery; 1 = status post cataract surgery)

General linear model (GLM) analysis of influence of lens status on IOP elevation showed, that it came slower to IOP spike in phakic eyes, with a peak IOP of 18.4 (an average, ± 0.7) mmHg at 6 months. In pseudophakic eyes IOP spike of 18.0 (± 0.5) mmHg could be observed at 6–8 weeks (p = 0.019, Fig. 4).

### Therapy

In total, 119 (70.8%) patients received a conservative therapy with IOP-lowering eye drops. There was no standard scheme for prescribing the eye drops, neither in our department nor at external doctor´s practices. Prescriptions included alpha agonists, beta blockers and carbonic anhydrase inhibitors. When the follow-up exams showed an IOP of ≤ 20 mmHg, we considered this as well regulated, and a conservative therapy could be discontinued.

Sufficient IOP regulation was achieved in 82 eyes (68.9%) treated with topical therapy. In 37 eyes (31.1%) with persistently elevated intraocular pressure, topical therapy had to be continued past the follow-up.

Surgical intervention was performed in 21 eyes with persistently elevated IOP and increase in cup-to-disc ration (CDR). Out of these 21 cases, we performed a cyclophotocoagulation in 14 eyes (8.3%), a filtering surgery in 3 eyes (1.8%) and we removed a steroidal drug implant – FA – from 4 eyes (2.4%). Steroidal drug implants were removed in 1 eye treated for a postoperative macular edema (25%), in 2 eyes treated for a diabetic macular edema (50%) and in 1 eye suffering from a retinal vein occlusion (25%).

28 eyes received no therapy (16.7%).

## Discussion

Common side effects of intraocular glucocorticoids may include postinjection infectious endophthalmitis, secondary ocular hypertension, or secondary steroid-induced open-angle glaucoma, rhegmatogenous retinal detachment, postinjection steroid-induced cataract, central serous chorioretinopathy, and toxic effects [27–30]. However, an increase in IOP is one of the most widely discussed topics in terms of its possible future implications, i.e. development of a secondary steroid-induced glaucoma. When compared to intravitreal injection, sub-Tenon application of TMC has a lower risk of IOP elevation due to a lower intraocular concentration and shorter duration of action [31, 32].

Literature describes a hypertensive ocular response in up to 50% of eyes that are injected with a corticosteroid [10, 33]. In our study, 57.7% out of all eyes with an IOP elevation had been treated with a steroidal drug monotherapy. According to the official summary of product characteristics, an IOP elevation of ≥ 10 mmHg from baseline occurred in 28% at any visit, while an IOL of ≥ 30 mmHg occurred in 15% of patients who were subjects of an observational study and received DXM. In our study, an IOP rise, defined as a single measurement of ≥ 25 mmHg [12], was documented in 39.3% of all eyes. Similarly, Rezkallah et al. [34] analyzed 494 eyes after an intravitreal injection of a dexamethasone-implant, out of which 32.6% presented with ocular hypertension (OHT). The main indications for treatment in their study were retinal vein occlusion, diabetic macular edema, postsurgical macular edema and uveitis. The SAFODEX study [12] showed that dexamethasone-implants, injected in 421 eyes for retinal vein occlusion, diabetic macular edema, postsurgical macular edema and uveitis, caused OHT in 28.5% of cases. In the ZERO study, conducted by Schmitz et al., less than 20% of the eyes after dexamethasone injections for retinal vein occlusion presented with elevated IOP.

As far as intravitreal injections with fluocinolone acetonide are concerned, main adverse effects include the risk of raised intraocular pressure, as well as cataract development [23]. According to ILUVIEN’s summary of product characteristics, IOL elevation is a very common adverse event with IOP > 25 mmHg at 21% and > 30 mmHg at 14% of patients in an observational study. 38% of subjects required an IOP-lowering medication, whereas 5.6% had surgery to lower IOP. In our study, 5 of 16 eyes (31.3%) with FA monotherapy had an elevated IOP which occurred after a median of 11 months. In the FAME study, IOP increased in 37.1% of eyes after receiving an FA-injection [23]. In comparison, Alfaqawi et al. [35] documented an IOP rise of ≥ 10 mmHg in 3 eyes (11%). Fallico et al. [36] reported that 27% of their subjects required IOP-lowering drops and 3% glaucoma surgery. Interestingly, 1.8% of our patients’ eyes underwent a filtering glaucoma surgery and 2.4% had a steroidal drug implant – fluocinolone acetonide in all cases – removed.

In an article by Tao et al. [28], about 40% of eyes developed a secondary OHT after an intravitreal triamcinolone therapy, whereas in our study 47.6% of eyes presented with ocular hypertension. Furthermore, sub-Tenon triamcinolone acetonide caused an IOP elevation in 23.1% of eyes. Handzel et al. [37] as well as Cardillo et al. [31] stated, that there was no significant rise in IOP after sub-Tenon triamcinolone acetonide, with Cardillo presenting no difference between the 2 types of triamcinolone acetonide application (intravitreal versus sub-Tenon).

The most common steroid combinations that resulted in an increase in IOP were intravitreal triamcinolone acetonide combined with dexamethasone (57.4%), dexamethasone with fluocinolone acetonide (56.8%), and sub-Tenon triamcinolone acetonide with dexamethasone (51.5%) (Table 3 and Fig. 1). These results let us suspect that therapy with dexamethasone, either as a monotherapy or in combination with another steroid, tends to increase IOP.

The rise of ocular pressure occurred on average after 7.5 months from the first steroid administration, with a median of 5.5 months. Subjects presented with an elevated ocular pressure of ≥ 25 mmHg after a minimum of 2 weeks and a maximum of 24 months. IOP rise was documented after a median of 1 month in case of intravitreal triamcinolone acetonide, with the first IOP elevation occurring after no more than 2 weeks. This correlates with results by Jonas et al. [30] who showed IOP rise already during the first week after the intravitreal injection. The longest mean increase of IOP with 11 months was observed after the application of fluocinolone acetonide. In comparison, Alfaqawi et al. [35] documented an IOP rise after a mean of 3 months after the application.

In our study, in 28 eyes (16.7%) with a single IOP rise, there was spontaneous normalization of IOP after discontinuation of steroidal therapy, without pressure-lowering topical therapy. Given a steroid induced elevated IOP, normalization is possible after simply stopping the steroidal therapy. However, to prevent structural and/or functional deterioration due to continuously increased IOP, patients suffering from SIOH, or SIG can be successfully managed with topical glaucoma medication, just the same as patients with primary open-angle glaucoma and ocular hypertension, without the impact of steroids. If these treatments fail to control the IOP, patients must undergo a traditional glaucoma surgical intervention, which is only required in less than 2% of cases [30]. According to the official summary of product characteristics of OZURDEX®, topical IOP lowering medication was applied in 42%, while surgical intervention for elevated IOP was needed in 1.2% of patients who were subjects of an observational study [38]. In the MEAD Study over 40% of eyes required a topical antiglaucoma therapy and 0.3% of eyes were treated with incisional glaucoma surgery [39]. Moreover, the Retisert-FA implant study reported an IOP elevation of ≥ 30 mmHg in more than 60% of eyes. As a result, one third of these eyes required glaucoma filtration surgery or explantation of the device [40]. Our study showed 168 eyes (39.3%) with an OHT after steroid injection, which were treated conservatively with topical antiglaucoma therapy (70.8% of eyes with elevated IOP). In comparison, 10% of eyes required topical glaucoma treatment after an IOP elevation in the EMR-study [41]. We considered an IOP to be well controlled when reaching an IOP of ≤ 20 mmHg under topical therapy, which was achieved in 82 eyes (48.8%) after 4.5 months on average. Li et al. [42] reached IOP control similarly by month 4. Surgical intervention was needed in 21 eyes (12.5%) with cyclophotocoagulation performed in 14 eyes (8.3%), glaucoma filtration surgery performed in 3 eyes (1.8%) and steroidal drug implant removal in 4 cases (2.4%). The IRSS Study showed that 2% of eyes required incisional glaucoma surgery [43], whereas in the EMR-study, this was only necessary in 0.3% [41].

As far as eyes with primary open-angle glaucoma are concerned, 35 out of 65 (53.8%) presented with an increased IOP, which is 20.8% out of all 168 eyes with an OHT and showed to be statistically significant with a p-value < 0.009. Similarly, in a study by Chin et al. [44], out of 13 eyes with pre-existing primary open-angle glaucoma, 6 developed OHT (46.2%). Moreover, Levin et al. [45] analyzed patients with a history of corticosteroid induced IOP elevation and historical non-responders after sub-Tenon corticosteroid injections. Their results showed that a higher rate of recurrent IOP elevation developed in historical responder eyes (44%) compared with 13% in non-responders.

Lens status might be a predictive factor for IOP elevation after steroid application, as IOP increased in 61% of phakic eyes, compared to 32.6% of pseudophakic eyes.

One of the major limitations to our study is the retrospective design which is the cause of some missing data. Therefore, prospective designs should be created to better research on and understand this topic. Moreover, as our clinic is located in a rural area with a poorly developed public transport system and some of the patients are multimorbid, the connection to a regular check-up, which is decisive for the success of filtering operations, was not given. For this reason, we preferred cyclophotocoagulation because of the reduced need for postoperative follow-up.

## Conclusions

In conclusion, every steroid therapy, regardless of the agent or indication, can lead to the development of ocular hypertension. The risk may vary according to the steroids and the treatment (e.g. monotherapy versus drug combination) used. Results of our study let us suspect that especially therapy with intravitreal dexamethasone, either as a monotherapy or in combination with another steroid, tends to increase IOP more than other steroids. An elevated IOP predisposes the patient to develop secondary steroid-induced hypertension, as well as secondary steroid-induced glaucoma with all of the disease’s sight-threatening aspects. Each patient should be intensively informed about the possible risks of therapy and attend regular IOP controls during follow-up to initiate an early intervention if necessary.
